# Carcass Characteristics and Beef Quality of Young Grass-Fed Angus x Salers Bovines

**DOI:** 10.3390/foods11162493

**Published:** 2022-08-18

**Authors:** Jingjing Liu, Marie-Pierre Ellies-Oury, Liselotte Pannier, Dominique Gruffat, Denis Durand, Faustine Noel, Bernard Sepchat, Isabelle Legrand, Sophie Prache, Jean-François Hocquette

**Affiliations:** 1 Institut National de Recherche pour l’Agriculture, l’Alimentation et l’Environnement (INRAE), Clermont-Ferrand, UMR1213 Herbivores, VetAgro Sup, 63122 Saint-Genès-Champanelle, France; 2 Bordeaux Sciences Agro, 1 Cours du Général de Gaulle, CS 40201, 33175 Gradignan, France; 3 Food Futures Institute, Murdoch University, Perth 6150, Australia; 4 Institut de l’Elevage, Caen Site, CEDEX 9, 14949 Caen, France; 5 Institut de l’Elevage, Limoges Site, CEDEX 2, 87060 Limoges, France

**Keywords:** farming system, crossbreeding, grass feeding, beef eating quality, fatty acid

## Abstract

To characterize carcass and meat attributes, such as beef eating quality in specific farming conditions, 31 young grass-fed crossbred Angus x Salers cattle in two farming systems (a mono-cattle system versus a mixed system with beef cattle and sheep) were used in this study. Three muscle cuts (striploin—*m. longissimus dorsi et thoracis*; bolar blade—*m. triceps brachii caput longum*; internal flank plate—*m. obliquus internus abdominis*) were used for consumer eating quality testing and striploin was used for panelist eating quality assessment, and objective measurements [Warner–Bratzler shear force (WBSF) and fatty acid (FA) and antioxidant contents]. Results indicated that the farming system had no impact on carcass characteristics or meat quality, but it tended to affect FA content, which is likely explained by between-system differences in animal maturity (assessed by ossification score). Animal gender had significant effects on three eating quality traits evaluated by untrained consumers, with higher flavor liking, overall liking, and overall meat eating quality (MQ4) scores in females than in males. Additionally, FA contents were correlated with sensory quality traits to varying extents: consumer-scored tenderness, flavor, and overall liking were mainly positively correlated with ω-3 and ω-6 polyunsaturated fatty acid (PUFA) contents, and panelist-evaluated tenderness and abnormal flavor were more positively correlated with total lipids, saturated fatty acid (SFA), and monounsaturated fatty acid (MUFA) contents. Overall, this study showed that specific grass-fed crossbred Angus x Salers cattle can produce lean meat rich in ω-3 PUFAs with a low ω-6/ω-3 ratio and with “better than average” beef eating quality.

## 1. Introduction

Novel livestock farming systems such as agroecological farming and sustainable intensification systems, have emerged in response to the growing ecological concerns about sustainable agriculture, especially in France [1]. Moreover, the beef industry is undergoing changes due to the increasing concern for animal welfare and environmental impact at the expense of intensive livestock production coupled with the increasing demand for beef of high nutritional and eating quality [2,3], also with the need to develop pathways that are context-specific, with more consideration given to the food origin, its quality, and the region where beef is produced [3].

Animal feeding and farming systems are evaluated as crucial factors of consumers’ meat-quality perception, and grass-fed beef is preferred by consumers over concentrate-fed beef, mainly based on visual (such as color) and credence (such as perceived traits) qualities [4,5]. Grass-fed beef products represent a premium niche market with certain additional value through better animal welfare, a cleaner environmental footprint, and enhanced nutritional quality in terms of a more favorable PUFA/SFA ratio (polyunsaturated fatty acid/saturated fatty acid ratio) and ω-6/ω-3 ratio (n-6 polyunsaturated fatty acid/n-3 polyunsaturated fatty acid ratio) [2,6]. Additionally, grass-fed products have been identified to be more tender, though containing less intramuscular fat [7], but may be associated with lower commercial quality (i.e., lower meat yield).

Furthermore, grass feeding typically results in leaner beef products [8], whereas beef products with higher intramuscular fat content tend to produce beef of superior eating quality. For instance, the marbling level of “abundant” corresponds to the grade of “prime” in the United States Department of Agriculture (USDA) beef-grading system. Specific crossbreeding with early-maturing breeds to enhance fat synthesis and deposition is a way to take advantage of grass feeding systems in order to produce meat of good eating quality [9]. Hardy breeds, such as Salers, are well adapted to the mountainous pasture environment, unlike more specialized beef breeds (i.e., Angus). The early-maturing Angus breed is more adapted to grass feeding and fattening than continental beef breeds [10]. As such, the animals used in this experiment were crossed from an early-maturing sire breed (Angus) and a hardy dam breed (Salers) [11] with the objectives of rearing cattle well adapted to the mountainous environment and grass-feeding system to potentially produce beef of good eating quality. Additionally, carcass characteristics and meat quality have been poorly studied within this specific crossbreed, and were therefore tested within the experiment.

Mixed systems with different species seem to be effective at enhancing animal performance [12] and optimizing agronomic outputs [13]. In mixed-species systems, reducing animal density of one species provides benefits for grass consumption and pasture utilization efficiency [14] and also for parasite control through the dilution of parasites [15]. In comparison with monospecific systems, a mixed sheep/beef cattle system has increased meat production due to higher animal weight gain [16]. However, few studies have investigated the effect of mixed systems with a principal focus on animal performance, such as live weight gain [17]. What is yet less clear is the impact of mixed systems on carcass characteristics and meat eating quality attributes of tenderness, juiciness, and flavor, as well as FA composition.

To better meet these objectives of novel livestock farming systems, two strategies were studied in the current experiment: (1) specific crossbreeding of beef animals was performed for better adaptation to grass feeding and for a better beef eating quality, and (2) a mixed-species system was introduced for integrated management of parasites and higher efficiency of grass utilization. There were two major aims of the present study: (1) to examine the carcass and meat-quality attributes of the Angus x Salers beef cattle; (2) to investigate the effect of farming system (the association of beef cattle and sheep within a mixed system) on beef carcass characteristics and meat-quality attributes.

## 2. Materials and Methods

### 2.1. Experimental Design

The present study was based on an experiment conducted on the Laqueuille site of the Herbipôle experimental unit from INRAE. All cattle used in this study were an Angus x Salers cross. There were two independent farming systems: a monospecific beef cattle system (MONO, n_cattle_ = 22) and a mixed system with beef cattle and sheep (MIX, n_cattle_ = 13, n_sheep_ = 66). In the mixed system, beef cattle co-grazed with sheep from April until the end of July 2019, after which cattle grazed before the sheep from August until mid-October 2019. Calves were weaned in October and fattened indoors with grass haylage. The grass and haylage used to feed all the animals were from natural organic pastures with a variety of grass species (results of botanical analysis of grasses are presented in Supplementary Materials). All males were castrated one week after birth. Allocated areas and livestock units (LU) were the same in both systems, the mixed system being characterized by 40% sheep LU and 60% bovine LU.

### 2.2. Carcass Grading and Muscle-Cut Collection

Thirty-one cattle—20 (10 females and 10 castrated males) from the mono system and 11 animals (6 females and 5 castrated males) from the mixed system—were slaughtered at a young age (around 14 months). All procedures were approved by the C2EA-02 Ethics Committee (APAFIS#1417-2015081011477291 v3 and APAFIS#24191-2015043014541577 v4). Animal carcasses were assessed according to the EUROP grid (conformation and fat scores were converted into a continuous 15-point scale) [18] and by a certified grader for ossification, marbling, and hump height following the MSA (Meat Standards Australia) standards [19]. Critical methods of carcass grading are presented in Supplementary Materials. The descriptive data are presented in Table 1.

Left and right striploins (*m. longissimus dorsi et thoracis*), bolar blade (*m. triceps brachii caput longum*) and two portions of the internal flank plate (*m. obliquus internus abdominis*) were collected from each carcass at 24 h postmortem (pictures of raw muscles and cuts are presented in Supplementary Materials). These muscle cuts were selected to represent a potential difference in eating quality based on previous work in beef [20]. All muscle cuts were vacuum packed and stored at 4 °C before being frozen and used for further quality testing and measurements. Two aging times were applied to the bolar blade, with 31 muscle cuts aged 5 days and similar 31 muscle cuts aged 14 days. Both striploins and both portions of the internal flank plate were all aged 14 days. As such, in total, 186 samples were collected for subsequent testing sessions, with 31 striploin samples, 62 bolar blade samples, and 62 internal flank plate samples used for untrained consumers, whereas the other 31 striploin samples were used for the trained panelists.

Not all data are reported in the results. Corresponding information is mentioned in the table titles or notes.

### 2.3. Physicochemical Analysis

Measurements of muscle lipid, fatty acid analyses, antioxidant and vitamin contents were conducted on a subsample of the striploin and are described in detail in Hamdi et al. (2018) [21].

#### 2.3.1. FA Composition

FA composition was determined by chromatographic analysis after transmethylation. FAs underwent methylation by mixing of boron trifluoride (BF3) and methanol 14%. Methyl esters of FAs were then extracted by hexane and recovered after centrifugation at 1000× *g* for 10 min at room temperature. The FA composition was determined by gas chromatography flame ionization (CPG/FID, Shimadzu, Kyoto, Japan) using an Omegawax 250 capillary column (30 m, 0.25 mm ID). Injector and detector temperatures were 230 °C and 250 °C, respectively. Temperature was increased by 5 °C/min. FAs were identified and quantified, and the results are expressed as content (mg/100 g muscle).

#### 2.3.2. Antioxidant Content

A subsample of 250 mg of striploin muscle was homogenized in 3 mL of phosphate buffer (50 mM, pH 7) with a polytron for 15 s at 17,000× *g*. The homogenate was centrifuged at 1200× *g* for 15 min at 4 °C. The supernatant obtained was filtered and stored at 80 °C for determination of the activities of superoxide dismutase (SOD), glutathione peroxidase (GPx), and catalase (CAT).

SOD activity was determined by measuring the ability to inhibit the autoxidation of pyrogallol by 50%. The absorbance of the final solution was measured at 420 nm and 25 °C by a Uvikon 923 double-beam spectrophotometer (Kontron Analysis Division, Zurich, Switzerland).

GPx activity was determined by measuring the rate of oxidation of NADPH. The absorbance of the final solution was measured by the Uvikon 923 double-beam spectrophotometer at 366 nm and 37 °C. GSH Px activity is expressed as mmol NADPH/min.mg protein.

CAT activity was determined by measuring the rate of decomposition of hydrogen peroxide (H_2_O_2_) to H_2_O and O_2_. The absorbance of the final solution was measured using the Uvikon 923 double-beam spectrophotometer at 240 nm and 20 °C. CAT activity is expressed as mmol H_2_O_2_/min.mg protein.

#### 2.3.3. Vitamin Content

Vitamin A was extracted after saponification and hexane extraction. The hexane phase was removed by evaporation with a stream of gaseous nitrogen. Then, the dry extract obtained was solubilized by 240 mL of tetrahydrofuran and 240 mL of dichloromethane: methanol (65 V/35 V). Vitamin E concentration was determined by UV spectrophotometry at 292 nm and quantified using high-performance liquid chromatography (HPLC; model 430, Kontron) with Kroma System 2000 software (Kontron Analysis Division).

#### 2.3.4. Color and pH

Color was measured on the striploin 2 h after excision from the carcass using a spectrophotometer (Konica Minolta CM-600d, Osaka, Japan) and expressed in CIE L*, a*, and b* units. Five measurements per muscle (randomly distributed over the muscle but avoiding connective tissue) were used to determine the meat color. Meat pH was measured on striploin using a portable pH meter at 24 h postmortem.

### 2.4. Untrained Consumer Testing

A total of 155 muscle cuts were prepared for untrained consumer testing. According to the MSA grilling protocol [22], each muscle cut was sliced into five 25 mm-thick steaks. Each steak was further cut in half after cooking and 10 portions from one muscle cut were served to consumers. Steaks were grilled on a Silex clamshell grill set to 200 °C for 2.5 min up to an internal temperature of 55–57 °C. Representative pictures with three muscle cuts before and after cooking are presented in Supplementary Materials. Each consumer evaluated in total seven steaks—a starter steak (striploin) followed by six testing steaks (from striploin, bolar blade and internal flank plate)—that were served based on a 6 × 6 Latin square allocation for diverse quality levels and balancing order effect across consumers. Each consumer scored tenderness, juiciness, flavor liking, and overall liking on a 100 mm scale, with the left side of the scale representing not tender, not juicy, extremely dislike, and the right side representing very tender, very juicy, and extremely like. Then, consumers were asked to assign each sample to one of four MSA quality grades [2 star (unsatisfactory), 3 star (good everyday), 4 star (better than everyday), and 5 star (premium)] that best described their overall evaluation of the sample. A total of 360 untrained French consumers were recruited to participate in one of the 6 testing sessions with 60 consumers per session.

### 2.5. Meat Preparation, Trained Panelist Eating Quality Evaluation, and WBSF Measurement

Striploin samples were thawed at 4 °C for 24 h and cut into two subsamples for eating quality and WBSF measurement.

A panel of 20 members evaluated each meat sample using the monadic test method. Before the eating quality evaluation, the panelists were trained to be familiar with the meat eating quality evaluation traits. The meat samples were cut into 15 mm-thick steaks, which were grilled on a double-sided grill at 300 °C for 1.15 min to reach an internal temperature of 55 °C. Then, each sample was cut into 5 portions of 15 × 20 × 20 mm and each portion was sliced into 4 pieces to be served to four different panelists. For 5 testing sessions, 25 portions from 5 animals were evaluated using a 5 × 5 Latin square allocation, whereas 30 portions from 6 animals were evaluated using a 6 × 6 Latin square in the last session. In total, 6 sessions with 31 samples from 31 animals were evaluated by 20 panelists. These 20 panelists participated in each session, and each panelist tested 31 samples. Six eating quality traits were used to describe meat quality: initial tenderness, overall tenderness, initial juiciness, overall juiciness, typical flavor, and abnormal flavor. Each eating quality trait was scored on a 10-point non-graduated scale from 0 (tough, dry, slight flavor) to 10 (very tender, very juicy, intense flavor).

WBSF measurement of the raw meat was measured using MTS Synergie 200 [23]. For each sample, 5 cores of 1 cm meat were cut perpendicularly to the fibers. The peak force was recorded for each meat piece and the shear force was finally determined for each sample based on the average of the 5 measurements.

### 2.6. Statistical Analysis

Statistical analyses were performed with R software (4.1.1) and IBM SPSS 25. To assess the effect of animal gender and farming system and their interaction on carcass characteristics and meat quality, a univariate analysis of variance was performed using a general linear model with the post hoc (Tukey HSD) test, including Bonferroni correction for pairwise comparisons. The significance level was determined at a Bonferroni-corrected *p* < 0.05. This method was used to examine the significant differences between means of raw data. Data from 14-day-aged striploin were used in this analysis.

In the specific case of data from untrained consumers, a linear discriminant analysis was performed [package: MASS, function: lda] to calculate the optimal weightings of the four eating quality attributes (tenderness, juiciness, flavor liking, and overall liking) to predict the final MSA quality grades (2 star, 3 star, 4 star, and 5 star) based on the combined meat eating quality score (MQ4). The methodology is exhaustively described in Watson et al. (2008) [22]. The current MQ4 was calculated according to the following equation: 0.36 × tenderness − 0.02 × juiciness + 0.29 × flavor liking + 0.37 × overall liking. The calculation of MQ4 weightings was based on raw data on all samples (10 portions for each muscle cut) tested by consumers.

To provide potential structure of the entire dataset with different dimensions and accurate inference in terms of correlations among all the animal, carcass, and meat quality attributes, principal component analysis (PCA) and Pearson correlation analysis with hierarchical cluster analysis (HCA) were conducted [package: factoextra, corrplot, ClustOfVar, function: fviz_pca, corrplot, hclustvar]. Data from 14-day-aged striploin were used in this analysis.

To comparatively assess the eating quality level of the current samples with those previously reported to define an overall average eating quality level, a European consumer-testing dataset from a wide range of experiments conducted containing a large diversity of cattle types and beef cuts [24] was utilized, with chi-squared tests used in this analysis. A total of 1550 samples from 155 muscle cuts, including all the muscle cuts and aging times, were used in this comparative analysis.

## 3. Results

### 3.1. Effect of Animal Gender and Farming System on Carcass Characteristics

Animal gender significantly affected carcass weight and ossification score (*p* < 0.001), with females having lower carcass weight and higher ossification score (Table 2). There was a significant interaction between gender and farming system on fat and muscle percentage based on hot carcass weight (*p* < 0.01): the MIX females showed higher fat percentage and lower muscle percentage than MONO females, but no difference was observed for males. However, neither animal gender nor farming system showed any significant effect on marbling and fat cover, hump height, carcass conformation, pH, or meat color (*p* > 0.05).

### 3.2. Effect of Animal Gender and Farming System on FA Content, Antioxidant Content, Vitamin Content and Eating Quality

The farming system displayed significant effects on some FA contents (Table 3). On average, MIX animals had higher contents of total FA, SFA, MUFA, and CLA. Animal gender had significant effects on beneficial FA ratio, with females having higher PUFA/SFA ratio and lower ω-6/ω-3 ratio than males (*p* < 0.05). On average, no effect of farming system was found on antioxidant content or eating quality evaluated by untrained consumers (Table 4), and animal gender had significant effects on three eating quality traits evaluated by untrained consumers, with higher flavor liking, overall liking, and MQ4 scores for females (*p* < 0.05) (Table 4).

### 3.3. Relationships between Animal and Carcass Characteristics and Meat Eating Quality Attributes

**Figure 1:**
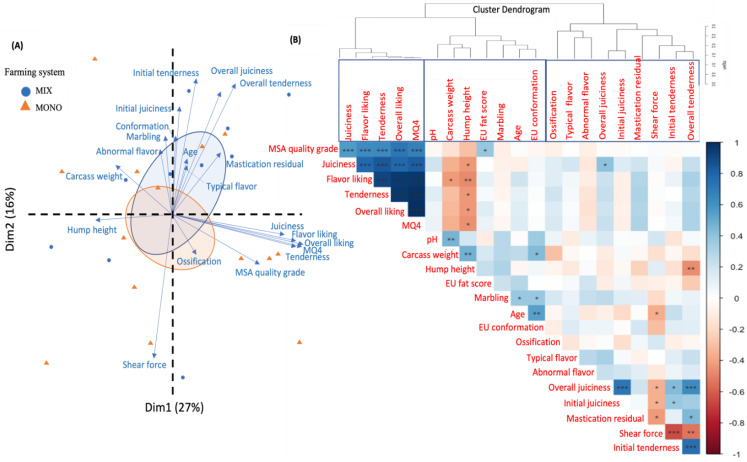
(**A**) Principal component analysis (PCA) and (**B**) Pearson correlation matrix with hierarchical cluster analysis (HCA) of animal and carcass characteristics and meat eating quality (14-day-aged striploin). *: *p* < 0.05; **: *p* < 0.01; ***: *p* < 0.001.

Animal age, marbling score, and conformation score were more related to eating quality perceived by panelists than consumers (Figure 1A). Carcass weight and hump height were more correlated with eating quality perceived by consumers. The two ellipses in Figure 1A represent the core area of the two farming systems, which are obviously not discriminated. Moreover, according to hierarchical cluster analysis (Figure 1B), carcass characteristics were clustered more closely to consumers’ evaluated eating quality than panelists’ evaluated eating quality.

The correlations between all the traits were confirmed by Pearson correlation coefficients. Some are indicated as being significant in Figure 1B, and others are partially presented below. Marbling was positively correlated with age (r = 0.38, *p* < 0.05). The ω-6/ω-3 ratio was negatively correlated with ossification score (r = −0.45, *p* < 0.05). CIE L* was negatively correlated with vitamin E content (r = −0.49, *p* < 0.05). Hump height had a negative correlation with all consumers evaluated eating quality traits, MQ4 score and overall tenderness evaluated by panelists (r = −0.46 to −0.50, *p* < 0.01). Similarly, carcass weight was negatively correlated with flavor liking (r = −0.43, *p* < 0.05). EU fat score was positively correlated with consumer-assessed MSA quality grades (r = 0.38, *p* < 0.05). One result of the current data is that consumer eating quality scores had no significant correlation with WBSF, and was only correlated with the panelists’ scores for juiciness (r = 0.4, *p* < 0.05). Tenderness and juiciness evaluated by panelists were negatively correlated with WBSF (r = −0.65, −0.36, *p* < 0.01). In addition, contents in SOD, some ω-3 (EPA, DPA), and some ω-6 (ARA) PUFA were positively correlated with consumers scored tenderness, flavor liking, and overall liking (r = 0.36 to 0.51, *p* < 0.05). Panelist-evaluated tenderness was positively correlated with the content of total lipids, SFA, MUFA, ω-6 PUFA, and certain ω-3 PUFA (EPA) (r = 0.37 to 0.46, *p* < 0.05) and negatively correlated with ω-6/ω-3 ratio (r = −0.43, *p* < 0.05). Panelist-evaluated juiciness was correlated with catalase content and ω-6/ω-3 ratio (r = 0.49, −0.45, *p* < 0.05). Flavor evaluated by panelists had no correlation with FA content, but abnormal flavor was positively correlated with the content of total lipids, SFA, C16:1n-7, C18:1n-9, C18:1 9trans, total MUFA, MUFA cis, total CLA, and CLA 9cis11trans (r = 0.37 to 0.44, *p* < 0.05) and negatively correlated with DHA content (r = −0.36, *p* < 0.05). Correlation analyses with FA contents were performed on data from 30 animals, since the abnormal FA content of one sample was not considered due to a sampling problem.

### 3.4. The Eating Quality of Angus x Salers Beef Evaluated by Consumers

To assess the eating quality level of the Angus x Salers beef samples (Table 5, line A), a European consumer-testing dataset (Table 5, line B) from a wide range of experiments conducted with a large diversity of European cattle types and beef cuts [24] was utilized in the comparative analysis. The data comprised 743 animals and 86,624 beef tests. This dataset was used as a means to reflect an average and diverse eating quality level (based on the four MSA quality grades), and if the eating quality level of the Angus x Salers beef is higher than that of the European beef samples, a “better than average” quality might be reached. For Angus x Salers beef samples, the highest proportion was assigned to 5 star (27% versus 11% for European dataset), followed by 4 star (30% versus 24% for European dataset) and 3 star (26% versus 39% for European dataset), while only 17% of Angus x Salers beef samples were assigned 2 stars (versus 26% for European dataset), these differences being significant (*p* < 0.001) (Table 5, lines A and B). Thus, for the Angus x Salers beef samples, fewer samples had low-quality grades (2 star and 3 star), and more samples have high-quality grades (4 star and 5 star).

For a more relevant comparison of eating quality scores (tenderness, juiciness, flavor liking, overall liking, and MQ4) of Angus x Salers beef samples with that of the European dataset, we used striploin and bolar blade samples only, since data on internal flank plates are not available in the European dataset. Indeed, the Angus x Salers beef samples had higher scores for all eating quality traits (Table 6).

## 4. Discussion

### 4.1. Carcass Characteristics

By comparison with young Angus or Salers bovines slaughtered over decades in the same processing plant and assessed with the same measurements [25], the Angus x Salers animals of this study were lean based on fat percentage (19.52% for Angus and 16.07% for Salers versus 15.35% for Angus x Salers). Furthermore, the 2.92% of total lipids of the current animals is generally considered lean [26]. Grass feeding and the young age of our animals are likely the main reasons for this leanness [27].

Differences in fat percentage based on hot carcass weight between the two systems were observed for females only. In general, animals reared and fattened on intensive feeding systems show a tendency to have higher intramuscular fat (IMF) content, with females likely depositing more IMF than males [28]. However, this is not always the case, e.g., likely due to the lower intake energy levels, no difference in IMF content between sexes of feral cattle was observed under feral conditions in Doñana National Park, Spain [29]. This may fit with our observations that fat cover and marbling remained unaffected by gender and farming system due to the low energy of the grass-based diet in the current study.

In addition, no differences were found in meat color between females and males or between the two farming systems, which confirms previous findings that different rearing managements had little or no impact on beef color [30]. It is somewhat notable that the current beef samples were likely to be dark based on the low values of lightness and redness (CIE L* = 31.3, a* = 14.0), in particular when comparing to dark-cutting beef [31]. This is in accordance with the fact that grass-fed/finished cattle tend to produce darker meat [32], with high pH caused by a lower muscle glycogen content at the time of slaughter. However, the possible interference of pH can be ruled out, since the current pH was generally compliant (<5.7) and was not correlated with L* value in the present analysis. Moreover, CIE L* and a* values of the current samples (from around 200-day grass-fed animals) were numerically lower than in a study conducted with *longissimus dorsi et thoracis* muscle from 199-day grass-fed Charolais and Limousin bulls that had CIE L* and a* values of 46.5 and 18.0 [33]. This suggests the current beef may indeed be dark. It was demonstrated that beef color is considered acceptable for consumers when a* values are ≥ 14.5 [34], the current beef being close to this threshold, which may indicate that the current meat color is not too dark to be unacceptable for the consumer. In addition, grass diets can significantly improve beef antioxidant contents, preventing the oxidation of oxymyoglobin to metmyoglobin, which leads to negative changes in meat color [35]. However, given the low number of correlations between meat color and antioxidant contents in the present study, the low value of CIE L* may be explained by the specific crossbreeding and/or insufficient blooming time of the beef samples, which is recommended to be longer [36], hence sufficient oxidation of pigment had not fully occurred.

### 4.2. Fatty Acid Profile

FA content was unaffected by the interaction of animal gender and farming system. However, the females had a beneficially higher PUFA/SFA ratio and a lower ω-6/ω-3 ratio than males, which is likely due to the association between animal maturity and FA deposition. Females were more physiologically mature than males based on ossification score, with the ω-6/ω-3 ratio being negatively correlated with ossification score (*p* < 0.05), indicating that more mature females tended to deposit more PUFA (especially ω-3 PUFA) than males. The farming system mostly affected the SFA and MUFA contents, with only MIX animals having higher levels of those FAs than MONO animals. This may also be attributed to different animal maturity. It is well established that grass feeding can significantly improve beneficial FA composition with higher ω-3 PUFA content and lower ω-6/ω-3 ratio [2]. Indeed, the current grass-based diet and crossbreeding strategy resulted in a low ω-6/ω-3 ratio (1.6 relative to the recommended target of <2–3) [2]. The contents of SFA and MUFA increase faster than the content of PUFA with increasing fatness, and consequently, the relative proportion of PUFA and the PUFA/SFA ratio decrease. Hence lean and late maturing breeds would have a higher PUFA/SFA ratio than early-maturing breeds when slaughtered at the same carcass weight [2]. This fact can support our finding that the PUFA/SFA ratio of current animals was 0.23, which can be considered moderate, since beef PUFA/SFA ratio is generally low, around 0.1, except for very lean beef, where the PUFA/SFA ratio can be easily reach the recommended values for human nutrition (>0.4) [26]. By comparison of the current FA composition with that of grass-fed beef [37], the current animals presented quite good ω-3 PUFA composition with high content of ALA (51.3 mg/100 g muscle relative to a range of 28.1–52.8 mg/100 g muscle), EPA (11.0 mg/100 g muscle relative to a range of 5.8–24.5 mg/100 g muscle) and DHA (4.3 mg/100 g muscle relative to a range of 1.5–4.2 mg/100 g muscle), despite the fact that these comparisons may depend on genetic makeup, breed, gender, age, and geographic location, which all affect FA compositions [27]. Consumption of ω-3 PUFA is linked to a variety of health benefits, notably in the prevention of cardiovascular diseases. However, the Western diet is largely deficient in ω-3 PUFA, with the actual daily intake achieving only up to half the recommended amount (estimated actual consumption in the Western diet: 0.9 g/d ALA, 137 mg/d EPA and 101 mg/d DHA; recommendations: 1.8 g/day ALA, 250 mg/d EPA, 250 mg/d DHA) [38]. Consumption of 70 g of the current Angus x Salers beef can provide around 7.2 mg EPA. High ω-3 PUFA content can benefit human health, but the oxidation of ω-3 PUFA would produce health-impairing factors. However, in comparison with the literature on grass-fed beef [27,39], the antioxidant contents of our Angus x Salers animals seem outside an optimal range. This might produce health-risk components from ω-3 PUFA oxidation.

### 4.3. Eating Quality

No effect of farming system was found on eating quality, while significant effects of gender were detected by consumers, with higher scores of flavor liking, overall liking, and MQ4 for females. As expected, panelist assessments of eating quality of the beef samples from Angus x Salers animals seem better than that of purebred Salers (i.e., the panel scores of overall tenderness, juiciness, and flavor were 7.5, 7.3, and 6.8 for striploin of the present Angus x Salers animals, 5.5, 5.6, and 5.6 for striploin of young Salers bulls, and 5.1, 5.7, and 5.9 for striploin of Salers cull cows [40]). Although data from different experiments are not comparable, a mean value greater than approximately 7 for all eating quality traits may indicate an average or greater eating quality for Angus x Salers animals, despite the abnormal flavor, which was evaluated as extremely high (close to 10). This fits with earlier observations, which showed that consumers prefer eating quality of grain-fed beef because grass-fed beef contains “abnormal” pastoral flavor characterized as “grassy”, “wild” and “barny” and lacks normal beef flavor (reviewed by Pogorzelski et al., 2022 [41]). In addition, aroma volatiles are related to FA composition [42] with correlations found between abnormal flavor scores from panelists and total lipids, SFA, MUFA, CLA, and some ω-3 PUFA (DHA) contents. The oxidation of ω-3 PUFA might be associated with the high abnormal flavor score [2]. In this study, most correlations between sensory traits from panelists and FA contents were positive. The only negative correlation was between abnormal flavor and DHA content. No significant correlation was found between sensory traits from panelists and PUFA content. These observations differ from the findings by Ellies-Oury et al. (2021), which indicated that with *longissimus thoracis* muscles from young Charolais bulls, sensory traits (juiciness, overall liking and flavor) evaluated by panelists were negatively correlated with PUFA proportions [43]. This may be due to different animal types and production systems, and also different FA units (content or proportion) used in the different studies. Moreover, we found that consumer-scored tenderness, flavor liking, and overall liking were positively correlated with some ω-3 (EPA, DPA) and some ω-6 (ARA) PUFA contents. It can be concluded from those findings that the correlations and/or relationships between sensory traits and FAs can be variable according to animal types and farming systems.

Most importantly, on average, the current beef samples had higher consumer eating quality scores and a higher proportion of samples were assigned to the higher MSA quality grades of 4 star and 5 star, with a lower proportion assigned to being unsatisfactory than the beef samples from the European dataset. In general, IMF highly contributes to beef eating quality with strong correlations (r = 0.74 to 0.88, *p* < 0.05) between IMF content and tenderness, juiciness, flavor liking, and overall liking being reported [44]. The contribution of marbling score to the variability in MQ4 score is up to 51% for *m. longissimus thoracis et lumborum* [45]. However, in the present study, no significant correlation was found between marbling score and eating quality scores assessed by both consumers and panelists. This absence of significant correlation may be somewhat explained by the low range of variability of marbling score, and a greater number of animals with a larger phenotypic range may be warranted. Furthermore, this may also be explained by the fact that other factors aside from marbling affect beef eating quality [46] and the marbling effect may have been overshadowed by these other factors. For instance, consumer scores are related to FA composition, mainly ω-3 and ω-6 PUFA (i.e., EPA, DPA, ARA) contents, whereas tenderness evaluated by panelists was more related to total lipids, SFA, and MUFA contents. Consistent with the literature [47], ω-6/ω-3 ratio had a negative correlation with tenderness and juiciness. In fact, given that the current animals were young and lean, specific relationships might exist between animal and muscle characteristics and eating quality traits. Indeed, beef eating quality is multifactorial determined, for instance, with various effects of marbling depending on muscle cut [45] or with a low effect of breed on eating quality [48].

The WBSF measurement was used additionally in this study to provide more repeatable measurements of tenderness. It provides also indications of the strengths of the connective tissue and myofibrillar structures [49]. In the study of Dransfield et al., (2003) [40] with three muscles aged 14 days from four breeds, three quality classes (lowest, intermediate, and highest) were derived based on panelists’ eating quality assessment, with WBSF values of raw meat from the highest to the lowest eating quality classes being 79.4 to 94.1 N/cm^2^. Our result of WBSF on raw meat (77.1 N/cm^2^) suggests that the tenderness of Angus x Salers beef might be in the tender category (the highest-quality class set by Dransfield et al. (2003) [40]). Consumer assessment of tenderness and instrumental measures of tenderness have been extensively studied and their correlations were generally weak [r = −0.19 [50], −0.26 [51]] in most studies, but sometimes can be higher (r = −0.72) [52]. A higher correlation between panelist-scored tenderness and WBSF (r = −0.82) has been reported [53]. Our results confirm a weak correlation (*p* > 0.05) between consumer-assessed tenderness and WBSF and a moderate but significant correlation between panelist-assessed tenderness and WBSF. Although WBSF measurement is not a good indicator of consumer perception of meat eating quality, since the former is not only related to mechanical force but also associated with the sensations generated by moisture and fat within meat, whereas panelist assessment of quality can be reconciled to a certain extent with WBSF. This is consistent with Perry et al. (2001) [54], who found that consumers had the ability to detect any improvement in eating quality, but the improvement cannot be measured by WBSF. Conversely, panelists could better discriminate tender and tough meat in accordance with WBSF. This can further support the finding of no correlation between consumers’ and panelists’ assessments of beef eating quality. Trained panelists and untrained consumers may have different emphases when tasting beef; for example, as panelists are more qualified and guided by more granular eating quality traits, they may intentionally focus on certain aspects that determine eating quality, such as abnormal flavor, which may be not noticed by untrained consumers. Although both methods have been found to be effective in assessing the eating quality of meat, this uncorrelated relationship is understandable given the different processing protocols (i.e., sample size and cooking temperature) and scales of the two methods. Moreover, the low number of animals and beef samples potentially with a low range of variability of eating quality can also explain these uncorrelated results.

## 5. Conclusions

This study demonstrated that young grass-fed crossbred Angus x Salers animals can produce beef of “better than average” eating quality with low intramuscular fat content, high ω-3 PUFA content, and a low ω-6/ω-3 ratio. This is of importance due to the paucity of research on grass-fed Angus x Salers animals. FA composition had correlations with beef eating quality in varying extents: consumer-scored tenderness, flavor liking, and overall liking were mainly related to ω-3 and ω-6 PUFA contents, and panelist-evaluated tenderness was more correlated with the contents of total lipids, SFA, and MUFA. The ω-6/ω-3 ratio was positively correlated with WBSF and negatively correlated with panelist-scored tenderness and juiciness.

To summarize, crossbreeding and especially the grass-feeding strategy could contribute together to good animal performances in terms of FA profile and meat eating quality. Although the association of beef cattle and sheep within a mixed system had very little or no impact on carcass or meat attributes, the findings of this study have important implications for future practices: (1) beef cattle and sheep mixed farming systems, which have agroecological benefits, can be implemented without undue penalty to meat quality; (2) Angus x Salers crossbreeding with a grass-feeding strategy is relevant due to the higher beneficial FA composition and above-average eating quality.

## Figures and Tables

**Table 1:** Unadjusted means, standard deviations, minimum and maximum values for animal age, carcass traits, meat pH, color, lipid content, FA content and FA index.

Traits	Mean	SD	Min	Max
Age (days)	422	16.8	386	458
HCW (hot carcass weight, kg)	226.6	17.4	190.2	254.2
CCW (cold carcass weight, kg)	226.0	17.5	190.2	254.2
Hump height (cm)	3.5	0.7	2	5
Ossification (100–590)	130	20.8	100	190
Marbling (100–1190)	240	56.4	160	350
pH	5.66	0.13	5.48	5.93
EU conformation (1–15)	8	0.5	7	8
EU fat score (1–15)	6	0.8	5	8
Fat % HCW ^1^ (%)	15.35	1.34	12.90	18.50
Muscle % HCW ^2^ (%)	66.57	1.38	63.80	69.10
CIE L* (lightness)	31.32	2.07	26.39	36.94
CIE a* (redness)	14.02	1.37	10.66	16.69
CIE b* (yellowness)	14.66	1.42	11.10	17.34
Total lipids ^3^	2.92	0.54	1.92	3.99
C18:3n-3 ^4^ (ALA ^5^)	51.30	10.16	33.89	73.35
C20:5n-3 (EPA ^6^)	11.04	3.95	6.26	21.02
C22:5n-3 (DPA ^7^)	20.11	4.62	11.96	27.93
C22:6n-3 (DHA ^8^)	4.30	4.40	0.00	12.7
PUFA/SFA ratio ^9^	0.23	0.05	0.14	0.37
ω-6/ω-3 ratio ^10^	1.60	0.18	1.26	1.97

^1^ Fat % HCW: fat percentage based on hot carcass weight [fat weight (kg)/hot carcass weight (kg) %]; ^2^ Muscle % HCW: muscle percentage based on hot carcass weight [muscle weight (kg)/hot carcass weight (kg) %]; ^3^ Total lipids unit: g/100 g muscle; ^4^ FAs [fatty acids] are expressed in content (mg/100 g muscle); ^5^ ALA: alpha-linolenic acid; ^6^ EPA: eicosapentaenoic acid; ^7^ DPA: docosapentaenoic acid; ^8^ DHA: docosahexaenoic acid; ^9^ PUFA/SFA ratio: polyunsaturated fatty acid/saturated fatty acid ratio; ^10^ ω-6/ω-3 ratio: n-6/n-3 PUFA ratio; ^3,5,6,7,8,9,10^ data based on 30 animals.

**Table 2:** Estimated marginal means of animal and carcass characteristics as affected by animal gender and farming system.

System (S)	MONO		MIX			Significance of F-Value		
Gender (G)	Female (n = 10)	Male (n = 10)	Female (n = 6)	Male (n = 5)	SEM ^1^	G	S	G × S
Age (day)	415	421	430	429	7.34	0.74	0.07	0.54
HCW ^2^ (kg)	208.9	238.8	225.9	238.7	5.23	<0.001	0.06	0.06
CCW ^3^ (kg)	208	238.5	224.8	238.3	5.19	<0.001	0.07	0.06
Hump (cm)	3.0	4.0	3.0	3.5	0.31	0.08	0.23	0.41
Ossification	140 ^a^	120 ^b^	150 ^a^	110 ^b^	6.58	<0.001	0.97	0.09
Marbling	230	250	250	220	26.1	0.75	0.82	0.33
pH	5.7	5.7	5.6	5.7	0.06	0.27	0.49	0.52
EU conformation ^4^	7	7	7	7	0.23	0.2	0.79	0.80
EU fat score ^4^	5	5	6	6	0.35	0.96	0.2	0.54
Fat % HCW ^5^	14.6 ^b^	15.1 ^b^	17.1 ^a^	15.2 ^b^	0.47	0.08	<0.01	<0.01
Muscle % HCW ^6^	67.3 ^a^	66.6 ^ab^	65.3 ^b^	66.6 ^ab^	0.55	0.5	<0.01	<0.01
CIE L*	32.2	31.1	30.5	31.0	0.93	0.73	0.25	0.29
CIE a*	13.4	14.4	14.5	13.9	0.61	0.70	0.60	0.15
CIE b*	14.3	14.9	14.8	14.8	0.66	0.64	0.78	0.62

^1^ SEM: standard error of the mean; ^2^ HCW: hot carcass weight; ^3^ CCW: cold carcass weight; ^4^ on a 15-point European grid; ^5^ Fat % HCW: fat percentage based on hot carcass weight [fat weight (kg)/hot carcass weight (kg) %]; ^6^ Muscle % HCW: muscle percentage based on hot carcass weight [muscle weight (kg)/hot carcass weight (kg) %].^a, b^ Within a row, means with different letters are significantly different (*p* < 0.05) between different groups.

**Table 3:** Estimated marginal means of FA content, antioxidant content, and vitamin content as affected by animal gender and farming system.

System (S)	MONO		MIX		Significance of F-Value			
Gender (G)	Female (n = 10)	Male (n = 9)	Female (n = 6)	Male (n = 5)	SEM ^1^	G	S	G × S
(g/100 g muscle)								
Total lipids	2.68	2.92	3.35	2.89	0.23	0.57	0.11	0.09
Total FA	2.47	2.56	3.12	2.77	0.24	0.54	<0.05	0.30
(mg/100 g muscle)								
C12:0	3.73	4.39	5.96	6.17	0.65	0.44	<0.001	0.68
C14:0	62.32	72.09	92.46	78.24	8.98	0.77	<0.05	0.13
C16:0	566.4	600.2	730.7	639.5	62.61	0.59	0.07	0.25
C18:0	437.4	426.7	525.7	472.1	50.9	0.46	0.13	0.63
Linear SFA	1130.3	1166.3	1432.1	1265.3	127.1	0.55	0.08	0.36
Branched SFA	34.99	37.54	45.33	42.16	3.97	0.93	<0.05	0.40
Total SFA	1165.3	1203.9	1477.5	1307.4	130.8	0.56	0.07	0.36
C16:1n-7	45.81	50.70	59.92	56.57	5.66	0.87	<0.05	0.40
C18:1n-9	686.4	715.4	894.1	788.2	72.96	0.54	<0.05	0.29
C18:1 9trans	9.15	9.43	12.32	10.57	1.07	0.43	<0.05	0.27
C18:1 10–11trans	50.90	56.66	69.90	57.89	6.91	0.6	0.09	0.14
MUFA cis	822.7	862.4	1067.5	949.2	86.23	0.60	<0.05	0.29
MUFA trans	102.51	111.00	137.03	119.92	12.45	0.69	0.05	0.24
Total MUFA ^2^	925.2	973.4	1204.6	1069.1	96.42	0.60	<0.05	0.27
C18:2n-6 (LA) ^3^	76.80	79.52	81.25	74.40	4.37	0.58	0.93	0.21
C20:4n-6 (ARA) ^4^	25.96	24.39	31.93	24.20	2.40	<0.05	0.17	0.14
PUFA n-6 LC ^5^	38.71	34.85	46.77	37.01	3.48	<0.05	0.09	0.33
PUFA n-6 trans	25.93	29.52	33.76	30.47	2.87	0.95	0.08	0.17
Total PUFA n-6	147.4	152.0	169.6	146.6	9.94	0.29	0.33	0.12
C18:3n-3 (ALA)	50.28	49.48	55.97	51.03	4.66	0.48	0.37	0.61
C20:5n-3 (EPA)	11.60	9.68	13.17	9.80	1.75	0.09	0.57	0.63
C22:5n-3 (DPA)	19.32	18.39	23.34	20.91	2.00	0.33	0.07	0.66
C22:6n-3 (DHA)	4.68	3.66	4.71	4.21	2.07	0.67	0.87	0.88
PUFA n-3	96.31	91.33	107.87	98.50	9.81	0.40	0.27	0.80
CLA ^6^ 9cis11trans	20.37	23.96	28.57	25.64	2.55	0.88	<0.05	0.15
Total CLA	24.07	27.07	31.61	27.95	2.87	0.89	0.09	0.18
Total PUFA	272.7	275.1	315.3	279.2	21.70	0.37	0.22	0.31
C16:0/C18:0	1.38	1.36	1.32	1.37	0.06	0.05	0.34	0.59
PUFA/SFA ratio	0.25	0.20	0.25	0.22	0.02	<0.05	0.49	0.58
ω-6/ω-3 ratio	1.53	1.74	1.47	1.68	0.07	0.001	0.31	0.96
C18:2n-6/C18-3n:3	1.51	1.65	1.45	1.54	0.08	0.09	0.24	0.72
CAT ^7^	1.84	2.04	2.20	2.32	0.19	0.32	0.06	0.80
GPx ^8^	0.04	0.04	0.38	0.34	0.01	0.76	0.48	0.78
SOD ^9^	3.24	3.07	3.16	3.45	0.24	0.76	0.46	0.25
Vitamin ^10^ A	0.03	0.03	0.04	0.04	0.01	0.31	0.23	0.47
Vitamin ^10^ E	2.53	2.49	2.66	2.15	0.19	0.10	0.52	0.17

^1^ SEM: standard error of the mean; ^2^ MUFA: monounsaturated fatty acid; ^3^ LA: linoleic acid; ^4^ ARA: arachidonic acid; ^5^ PUFA n-6 LC: long-chain PUFA; ^6^ CLA, conjugated linoleic acid; ^7^ CAT, catalase (µmol H_2_O_2_ consumed/min per mg protein); ^8^ GPx, glutathione peroxidase (µmol NADPH/min per mg protein); ^9^ SOD, superoxide dismutase (IU/mg protein); ^10^ vitamin unit: µg/g tissue. All data used in this table are from 14-day-aged striploin. FA content results are presented for data from 30 animals, since the abnormal FA content of one sample from one male animal was not considered, due to a sampling problem.

**Table 4:** Estimated marginal means of eating quality scores evaluated by untrained consumers and trained panelists and WBSF as affected by animal gender and farming system.

System (S)	MONO		MIX			Significance of F-Value		
Gender (G)	Female (n = 10)	Male (n = 10)	Female (n = 6)	Male (n = 5)	SEM ^1^	G	S	G × S
Cut—striploin	Consumer testing							
Tenderness	60.99	47.63	59.86	51.5	6.78	0.07	0.81	0.66
Juiciness	62.08	52.54	55.70	57.44	3.75	0.23	0.82	0.09
Flavor liking	62.35	48.72	58.64	53.7	5.27	<0.05	0.89	0.34
Overall liking	60.45	48.69	58.95	50.84	5.52	<0.05	0.95	0.70
MQ4 ^2^	61.17	48.24	59.25	51.78	5.83	<0.05	0.90	0.49
Cut—striploin	Panelist testing							
Initial tenderness	7.45	7.58	7.76	7.41	0.23	0.58	0.71	0.22
Overall tenderness	7.56	7.41	7.73	7.54	0.27	0.44	0.51	0.93
Juiciness	7.38	6.99	7.27	7.49	0.31	0.74	0.45	0.25
Initial juiciness	6.92	7.08	6.92	7.04	0.27	0.54	0.93	0.93
Typical flavor	6.80	6.75	6.98	6.91	0.25	0.77	0.43	0.97
Abnormal flavor	9.67	9.71	9.75	9.66	0.09	0.78	0.86	0.43
Mastication residual	8.06	7.76	8.25	7.91	0.32	0.24	0.54	0.94
WBSF (N/cm^2^)	84.01	78.26	67.95	72.25	8.05	0.92	0.12	0.46
Cut—internal flank plate	Consumer testing							
Tenderness	63.12	69.09	65.39	67.53	4.74	0.32	0.93	0.64
Juiciness	58.70	65.51	60.92	62.01	4.25	0.28	0.86	0.43
Flavor liking	63.12	68.73	60.75	66.57	3.53	0.07	0.45	0.97
Overall liking	62.10	67.50	61.39	66.85	4.05	0.12	0.84	0.99
MQ4	62.84	68.47	62.55	67.10	4.01	0.14	0.81	0.87
Cut—bolar blade	Consumer testing							
Tenderness	70.05	63.82	62.42	64.50	4.74	0.53	0.33	0.26
Juiciness	70.14	64.00	63.58	69.53	3.85	0.98	0.88	0.07
Flavor liking	71.70	66.22	63.22	68.63	3.70	0.99	0.34	0.09
Overall liking	71.34	64.98	63.11	67.10	4.44	0.75	0.42	0.18
MQ4	71.35	64.94	62.85	66.57	4.25	0.71	0.35	0.17

^1^ SEM: standard error of the mean; ^2^ MQ4: combined meat eating quality score, which is used to describe the overall eating experience of consumers based on the perception of tenderness, juiciness, flavor liking, and overall liking. All data used in this table were from 14-day-aged beef samples.

**Table 5:** Distribution of beef samples to different MSA quality grades by consumers based on the present database versus the European database utilized.

Samples	Consumer-Assigned MSA Quality Grade				Chi-Squared Test
Dataset	2 star	3 star	4 star	5 star	*p*
	n ^2^ (% ^3^)	n (%)	n (%)	n (%)	
Angus x Salers ^1^ (A)	271 (17%)	401 (26%)	462 (30%)	412 (27%)	<0.001
EU dataset (B)	22,686 (26%)	33,809 (39%)	20,430 (24%)	9699 (11%)	

(A) 1546 beef samples of striploin, bolar blade and internal flank plate from the present study (1550 beef samples from 155 muscle cuts were tested by the current French consumers, 4 consumer data missed); (B) 86,624 beef samples from 22 muscle cuts were tested by consumers from France, Poland, Ireland, and Northern Ireland. ^1^ Angus x Salers: the present dataset; ^2^ n: the number of the samples tested by consumers in the corresponding category; ^3^ %: the proportion of samples of each MSA quality grade in all the samples of each dataset. In MSA consumer testing, each beef sample was divided into 10 portions and each portion was evaluated by one consumer. Then, the consumer was asked to assign each sample to one of the four MSA quality grades [2 star (unsatisfactory), 3 star (good everyday), 4 star (better than everyday), and 5 star (premium)] best describing their overall evaluation of the sample.

**Table 6:** Consumer scores on meat eating quality traits based on the present database versus the European database utilized.

Cut	Dataset	n	Tenderness	Juiciness	Flavor Liking	Overall Liking	MQ4
Bolar blade	Angus x Salers ^1^	31	66.19 ^a^	66.79 ^a^	67.79 ^a^	67.01 ^a^	66.91 ^a^
	EU ^2^	54	50.36 ^b^	56.98 ^bc^	55.60 ^b^	54.29 ^b^	52.44 ^c^
Striploin	Angus x Salers ^3^	31	54.94 ^ab^	57.01 ^bc^	55.84 ^b^	54.82 ^b^	55.36 ^bc^
	EU ^4^	326	52.74 ^b^	53.61 ^c^	55.58 ^b^	54.58 ^b^	53.69 ^c^
		SEM	2.99	2.52	2.28	2.49	2.48
		*p*	<0.001	<0.001	<0.001	<0.001	<0.001

^1^ Angus x Salers: 31 14-day-aged bolar blade samples from the present dataset; ^2^ EU: 54 blade samples from 45 young animals of 3 breeds; ^3^ Angus x Salers: 31 14-day-aged striploin samples from the present dataset; ^4^ EU: 326 striploin samples from 267 young animals of 9 breeds. Internal flank plates were not analyzed in the EU dataset, so the comparison was made based only on striploin and bolar blade. ^a, b, c^ Within a row, means with different letters are significantly different (*p* < 0.05) between different groups.

## Data Availability

Data are available on request to the authors.

## Supplementary Materials

The following supporting information can be downloaded at: https://www.mdpi.com/article/10.3390/foods11162493/s1, Supplementary Materials File S1: I—Animal feeding, management and performances; II—Critical description of carcass grading, consumer test and sample preparation and Quality Assurance. References [19,22] are cited in Supplementary Materials.
