# Supplementary material for: Carcass Characteristics and Beef Quality of Young Grass-Fed Angus x Salers Bovines
Source: Foods. 2022 Aug 18;11(16):2493. doi: 10.3390/foods11162493 (PMC9407266; doi:10.3390/foods11162493)
Supplement: Supplementary file 1 [file foods-11-02493-s001.zip › foods-1846735-supplementary.pdf]

Supplementary Material

I - Animal feeding, management and performances

The present study was based on an experiment conducted on the Laqueuille site of the Herbipôle experimental unit from INRAE.
Animals were born in the experimental farm with an average body weight of 43.4 kg (SD: 4.59 kg). They were weaned at 284 days of age with an average body weight of 315.1 kg (SD: 33.32 kg). Average daily gain until weaning was 1.1 kg/day (SD: 0.09). Body weight was 333.2 kg (SD: 34.56) when animals entered the finishing period at the age of 421.9 days (SD: 16.77). Mean age and mean weight at slaughter were 422.0 days (SD 16.78) and 448.9 kg (SD: 28.52), respectively. Average daily gain during the whole life of animals was 1.0 kg/day (SD: 0.07).

Animals were grazing from April until mid-October 2018. Calves were weaned in October and fattened indoors with grass haylage. The grass and haylage used to feed all the animals were from natural organic pastures with a variety of grass species.

Results of botanical analysis of the different meadows used for this experiment are the following:

**Main species**	**Proportion**
**Meadow number 1**	
Taraxacum sect. Taraxacum	14,5%
Anthoxanthum odoratum L., 1753	12,3%
Agrostis capillaris L., 1753	12,3%
Dactylis glomerata L., 1753	10,4%
Holcus mollis L., 1759	10,4%
Trifolium repens L., 1753	10,4%
Schedonorus arundinaceus (Schreb.) Dumort., 1824	4,5%
Poa pratensis L., 1753	4,0%
**Meadow number 2**	
Agrostis capillaris L., 1753	23,4%
Taraxacum sect. Taraxacum	14,7%
Dactylis glomerata L., 1753	10,6%
Trifolium repens L., 1753	10,6%
Anthoxanthum odoratum L., 1753	8,3%
Lolium perenne L., 1753	8,3%
Poa pratensis L., 1753	6,1%
**Meadow number 3**	
Dactylis glomerata L., 1753	13,8%
Geranium sylvaticum L., 1753	8,8%
Taraxacum sect. Taraxacum	8,8%
Agrostis capillaris L., 1753	6,9%

Anthoxanthum odoratum L., 1753	6,9%
Trifolium repens L., 1753	6,9%
Holcus lanatus L., 1753	5,0%
Lolium perenne L., 1753	5,0%
Lolium multiflorum Lam., 1779	5,0%
Plantago lanceolata L., 1753	4,7%
**Meadow number 4**	
Trifolium repens L., 1753	14,2%
Agrostis capillaris L., 1753	8,8%
Taraxacum sect. Taraxacum	8,7%
Poa pratensis L., 1753	8,5%
Rumex acetosa L., 1753	8,0%
Anthoxanthum odoratum L., 1753	6,6%
Phleum pratense L., 1753	6,1%
Bromus hordeaceus subsp. hordeaceus L., 1753	5,3%
Plantago lanceolata L., 1753	5,1%
Dactylis glomerata L., 1753	4,7%
**Meadow number 5**	
Bistorta officinalis Delarbre, 1800	22,1%
Anthriscus sylvestris (L.) Hoffm., 1814	17,4%
Trifolium repens L., 1753	12,2%
Bromus hordeaceus subsp. hordeaceus L., 1753	8,0%
Taraxacum sect. Taraxacum	8,0%
Phleum pratense L., 1753	7,3%
Rumex acetosa L., 1753	5,2%

No chemical composition of the animal diet can be provided since animal feeding is based on pastures (except botanical composition above).

II - Critical description of carcass grading, consumer test and sample preparation and Quality Assurance

A full description of our critical methods is provided below. Most of them have been previously validated following Quality Assurance rules as described in the indicated publications.

The main critical methods are for carcass grading which should follow AUS-MEAT chiller assessment standards. AUS-MEAT is an external organism for the accreditation of carcass graders. Carcass graders have previously followed a training of more than 10 days and have passed successfully different exams to be accredited. Repeated exams using a software on a specific computer (called OsCap) should be performed successfully every two months to ensure reproducibility of carcass grading.

1. **Carcass grading**

   Carcass grading was conducted by an AUS-MEAT certified carcass grader, all the assessments were carried out in strict accordance with AUS-MEAT chiller assessment standards (AUS-MEAT, 2010). The technical guidelines and quality assurance issued by AUS-MEAT are indicated below.

AUSTRALIAN BEEF CARCASE EVALUATION

Chiller Assessment Language

Chiller Assessment was developed to enable AUS-MEAT accredited Enterprises to assess, grade or class carcases using a uniform set of standards under controlled conditions. Chiller Assessment provides a means of describing meat characteristics and of classifying product prior to packaging. These characteristics include the colour of meat and fat, the amount of marbling, eye muscle area, the rib fat and the maturity of the carcase.

Assessments are made by qualified assessors and results are allocated to the carcase and provide a means of (carcase) selection according to individual contract specifications.

The AUS-MEAT Chiller Assessment Language is only available to AUS-MEAT accredited Enterprises, their clients and suppliers.

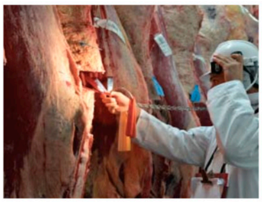

MARBLING

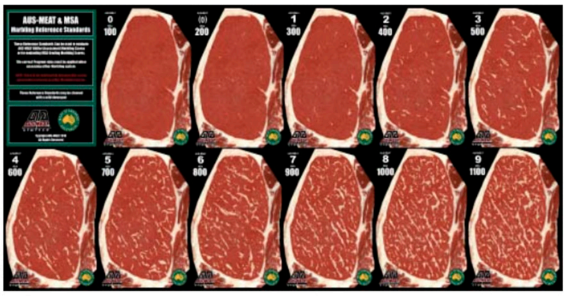

Marbling is the fat that is deposited between muscle fibres of the M. longissimus dorsi muscle. Marbling is assessed and scored against the AUS-MEAT / MSA Marbling reference standards.

The AUS-MEAT Marbling system provides an indication of the amount of marbling in beef. The MSA marbling system provides an additional indication of distribution and piece size.

Marbling is an assessment of the chilled carcase and scored by comparing the proportion of marble fat to meat at the surface of the assessment site which lies within the M. longissimus dorsi boundary.

Marbling may be assessed at any ribbing site from 5th-13th rib. The rib at which the measurement was performed must be nominated in company records.

RIB FAT MEASUREMENT

SUBCUTANEOUS

Subcutaneous Rib Fat measurement is a measurement in millimetres of the thickness of subcutaneous fat at a specified rib.

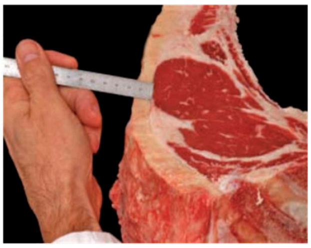

TOTAL

Total Rib Fat measurement is a measurement in millimetres of the thickness of subcutaneous fat and intermuscular fat at the specified rib.

CARCASE MATURITY

Maturity is an estimation of the development of a beef carcase determined by the degree of ossification of the dorsal spinous processes of the vertebrae, the fusing of the vertebrae, and the shape and colour of the rib bones.

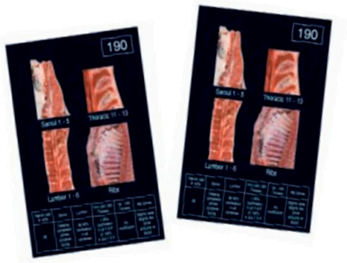

Maturity images depict MSA Standards

EYE MUSCLE AREA (EMA)

EMA is the area of the surface of the M. longissimus dorsi at the ribbing site and is calculated in square centimetres. EMA may be measured at the 10th, 11th, 12th or 13th rib.

EMA is measured manually using a plastic grid.

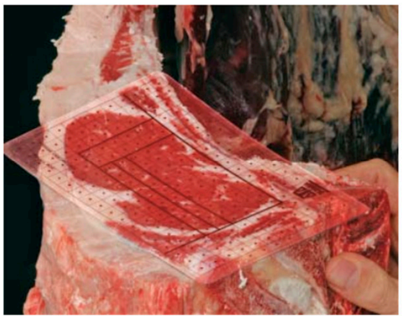

How to become a competent Chiller Assessor

To pass this course and become a competent chiller assessor – you must meet certain criteria. These are divided into a practical component and a theory component. Below are the assessments you are required to complete

(i) Chiller assessment theory assessment (70%)

There will be an open book multiple choice assessment that may cover any of the learning outcomes detailed in this course. The time frame for this exam is 1 hour.

(ii) Chiller assessment technique assessment (100%)

You will be practically assessed on your time at the course for all practical techniques required by a competent chiller assessor.

(iii) Achieve the AUS-MEAT Chiller Assessment correlation standard

Provided all the above have been successfully completed and to commence chiller assessment you must obtain the Chiller Assessment correlation standard. This is done by completing the necessary correlations using the OsCap™ correlation program back on your plant. You must then continue to maintain your currency for each of the required assessments on an 8-weekly cycle.

When is a participant deemed competent?

A participant is deemed competent at Chiller Assessment if they can correlate 10 runs on the OsCap™ system with 80% success. However, runs 9 & 10 have to be correct. This is for Marbling, Meat Colour and Fat Colour. There must be 2 successive runs using the OsCap™ system for Maturity, Rib fat Total Rib fat and Eye Muscle Area. The participant must at all times use the correct techniques when assessing product.

Non-competent

A participant is deemed non-competent if:

They are unable to demonstrate correlation accuracy using the OsCap™ system. A participant who fails to use the correct techniques will be deemed non-competent. A participant who fails the theory criteria will be deemed non-competent.

©Copyright, AUS-MEAT Limited. All rights reserved.

Onsite Correlation and Practice Program (OsCap™)

What is OsCap?

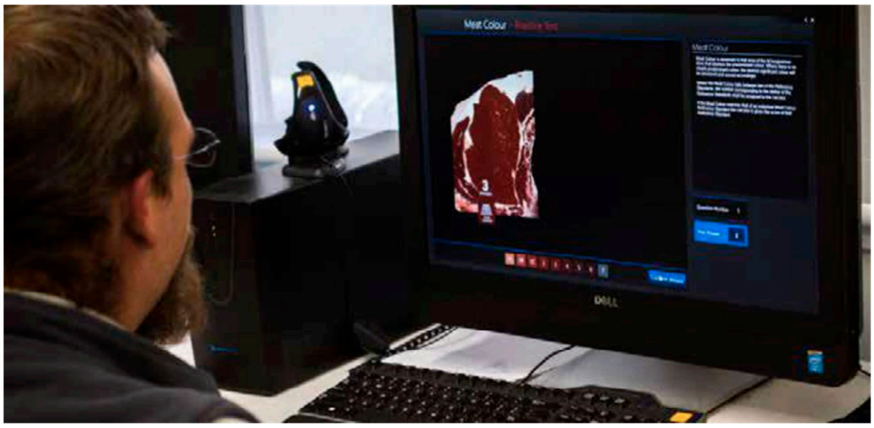

The AUS-MEAT Chiller Assessment program is underpinned by an Approved Quality System independently audited by AUS-MEAT. The integrity of the Chiller Assessment program is based upon;

- Training, qualification and currency status of Chiller Assessors and MSA Graders
- Correct use of the Chiller Assessment language and procedures for assessment;
- Maintenance and correct use of Chiller Assessment equipment;
- A regular cycle of Chiller Assessment Correlations. OsCap is the worlds first objective system for training and correlating for AUS-MEAT Chiller Assessors and MSA Graders.

The OsCap provides a flexible and effective method for training, on plant correlations and the continuous option to practice.

©Copyright, AUS-MEAT Limited. All rights reserved.

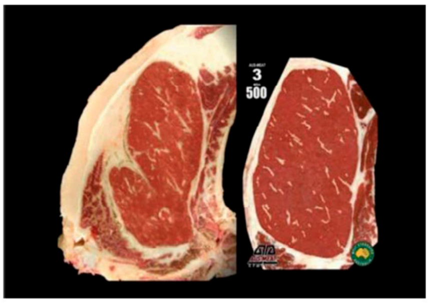

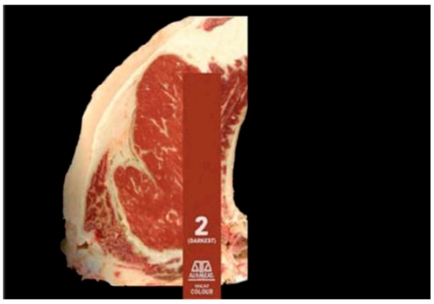

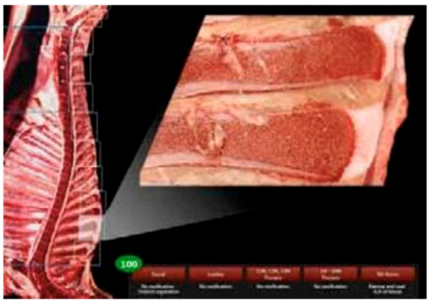

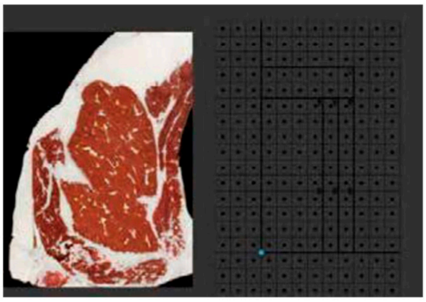

OsCap provides correlation and practice for:

- Chiller Assessment
- High Marbling Endorsement (for Assessors who grade Marbling above score 6)
- Maturity (ossification)
- Subcutaneous Rib Fat
- Total Rib Fat
- Eye Muscle Area
- MSA Marbling
- Hump Height
- Fat Distribution and Hide puller damage

©Copyright, AUS-MEAT Limited. All rights reserved.

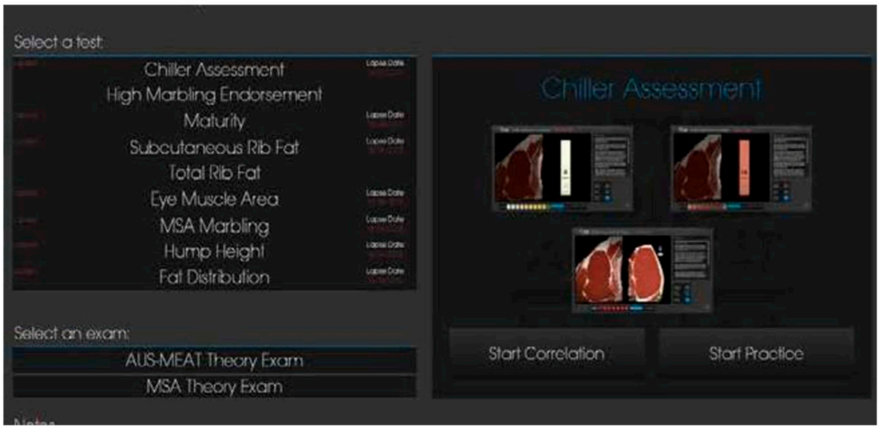

OsCap also includes a practice mode that can be used for in house training purposes such as prior to attending a Chiller Assessment training course or simply to allow the Assessor to tune more closely to the standard, and in practice mode the system provides instant feedback for the Assessor, so that the correct scores are reinforced.

OsCap™ also has a reporting system that will automatically provide records of correlations for Enterprise Quality Assurance systems.

The introduction of OsCap as the method of correlation positions Australia as having the only beef carcase assessment system which is correlated against an objective computer calculated standard.

OsCap™ has been developed in a co-operative approach with Meat Standards Australia and the correlation system incorporates standards and assessment criteria for the MSA grading.

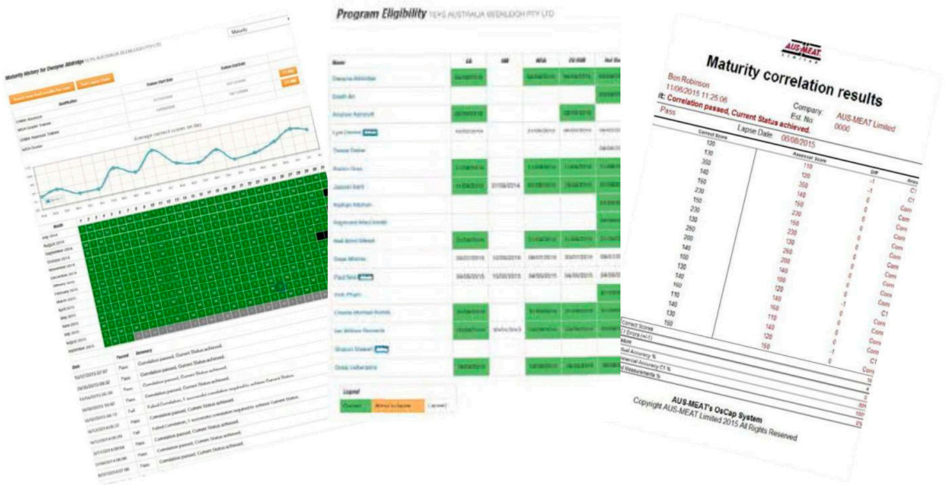

©Copyright, AUS-MEAT Limited. All rights reserved.

OsCap™ correlation requirements

Marbling, Meat Colour, Veal Colour and Fat Colour

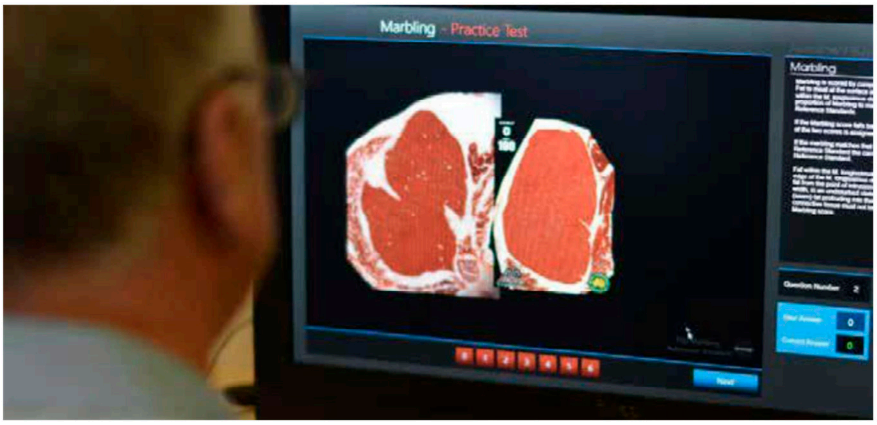

AUS-MEAT Marbling, Meat Colour, Veal Colour and Fat Colour

(a) Actual Accuracy

There must be agreement between the standard and the person being assessed on 70% of the sample.

For example, agreement must be reached on at least 14 out of 20 assessments in the sample.

(b) Commercial Accuracy

1. Where the Standard and the person being assessed vary, there must not be more than 10% of assessments that show a (commercial) variance of more than ± 1 score.
2. No assessment may vary by more than ± 3 scores.

For example, in a 20 carcase sample, 14 assessments must be the same, 4 may vary by ± 1 score, and the remaining 2 assessments may vary by ± 2 or ± 3.

Definitions of scores

Actual Accuracy is a score which is the same as the correct score.

Commercial Accuracy (C1) is a score which is +/-1 from the correct score.

Commercial Variance (C2/3) is a score which is +/-2 or +/-3 from the correct score.

Failed Measurement is a score which is more than +/-3 from the correct score.

It is important that you analyse the data accurately during the course as this information will be used to assess your progress. The trainer will be checking your data sheets after each run.

©Copyright, AUS-MEAT Limited. All rights reserved.

MSA Marbling

(a) The variation between the assessment and the standard must not vary by more than one (1) score step on more than 30% of the sample.

One (1) score step is defined as ±50 of the Standard.

(b) No assessment may vary by more than three (3) score steps.

Three (3) score steps is defined as ±110

For example, in a 20 carcase sample, 14 assessments must be within one (1) score step (±50). The remaining 6 must be within two (2) score steps (±100)

©Copyright, AUS-MEAT Limited. All rights reserved.

AUS-MEAT Carcase Maturity Chart

Score	Approx. Age in Months	Sacral Vertebral Characteristics		Lumbar Vertebral Spinous Process (S.P.) Characteristics	11th-13th Thoracic Spinous Processes (S.P.) Characteristics	1st-10th Thoracic Vertebral Spinous Process (S.P.) Characteristics	Rib Bone Characteristics
		Capping Characteristics	Vertebra Characteristics				
**100**	9	No Ossification of cartilage.	1) Distinct separation of vertebra. 2) Red, soft.	No Ossification Red, soft.	No Ossification Red, soft.	No Ossification Very red chine. Soft	Narrow & oval. Lot of blood.
**110**	10	Cartilage capping has started. 10%-20%	Distinct separation of vertebra.	No Ossification	No Ossification	No Ossification Red chine.	Slightly narrow. Slightly oval. Lot of blood.
**120**	13	Cartilage capping 30%-40%	Vertebral gaps starting.	No Ossification	No Ossification	No Ossification Red chine.	Slightly wide. Slightly flat. Lot of blood.
**130**	15	Advanced capping 50%-70%	Vertebral gaps closing, some separation still visible.	No Ossification	No Ossification	No Ossification Red chine.	Slightly wide. Slightly flat. Moderate blood.
**140**	18	Advanced capping 80%-90%	Vertebral gaps closing, some separation still visible.	No Ossification or minor spots of Ossification in one or 2 S.P.	No Ossification	No Ossification	Slightly wide. Slightly flat. Moderate blood.
**150**	20	Capping completed but some cartilage visible.	Vertebral gaps closing, some separation still visible.	No Ossification or minor spots of Ossification in some S.P.	No Ossification	No Ossification	Slightly wide. Slightly flat. Moderate amount of blood.
**160**	22	Capping completed but small amounts of cartilage visible.	Vertebral gaps closing, some separation still visible.	10%-20% Ossification in some S.P.	No Ossification	No Ossification	Slightly wide. Slightly flat. Moderate amount of blood.
**170**	24	Capping completed. Almost complete fusing.	Vertebra almost totally fused.	30%-40% Ossification	No Ossification	No Ossification	Slightly wide. Slightly flat. Small amount of blood.
**180**	27	Capping completed. Almost complete fusing.	Vertebra almost totally fused.	50%-70% Ossification in all S.P.	No Ossification or minor spots of Ossification in 1 or 2 S.P.	No Ossification	Slightly wide. Slightly flat. Small amount of blood.
**190**	29	Capping completed.	Vertebra almost totally fused.	80%-90% Ossification in all S.P.	Less than 25% Ossification in all 3 S.P., or 100% in any 1 S.P.	No Ossification	Slightly wide. Slightly flat. Small amount of blood.
**200**	30	Complete fusing.	Vertebra fused.	Almost complete Ossification.	>25% Ossification in all 3 S.P., or 100% in any 1 S.P.	Minor Ossification. Slightly red chine.	Slightly wide. Moderately flat. Traces of blood.
**230**	34	Complete fusing	Vertebra fused.	Almost complete Ossification.	30%-40% Ossification in all 3 S.P., or 100% in any 1 S.P.	Minor Ossification in some of the first 6 thoracic vertebrae. 10-20% in 7th - 10th	Slightly wide. Moderately flat. Traces of blood.
**250**	36	Complete fusing.	Vertebra fused.	Almost complete to complete Ossification.	>50% Ossification in all 3 S.P., or 100% in any 1 S.P.	10%-20% Ossification in some of the first 6 thoracic S.P. 30%-50% in 7th-10th S.P.	Moderately wide. Moderately flat. Traces of blood.
**280**	40	Complete fusing.	Vertebra fused.	Complete Ossification.	>70% Ossification in all 3 S.P., or 100% in any 1 S.P.	>30% in the 1st - 10th vertebrae.	Moderately wide. Moderately flat. Traces of blood.
**300**	42	Complete fusing.	Vertebra fused.	Complete Ossification.	80%-90% Ossification in all 3 S.P., or 100% in any 1 S.P.	> 30% Ossification in some of the first 6 thoracic vertebrae. 50%-70% in 7th-10th S.P.	Moderately wide. Moderately flat. Traces of blood.
**350**	57	Complete fusing. White.	Vertebra fused.	Complete Ossification.	Almost complete to complete Ossification.	40%-80% Ossification involving all S.P.	Wide & flat. No blood.
**400**	72	Complete fusing. White, extremely hard.	Vertebra fused.	Complete Ossification. White, hard.	Complete Ossification. Outlines barely visible.	Almost complete Ossification. Outline plainly visible.	Wide & flat. No blood.
**500**	96	Complete fusing. White, extremely hard.	Vertebra fused.	Complete Ossification. White, extremely hard.	Complete Ossification. White, extremely hard.	Complete Ossification. Outlines barely visible. White, hard.	Wide & flat. No blood.
**590**		Complete fusing.	Vertebra fused.	Complete Ossification.	Complete Ossification.	Complete Ossification, white chine	Wide & flat. No blood.

OsCap™ correlation requirements

Maturity, Eye Muscle Area, Subcutaneous and Total Rib Fat

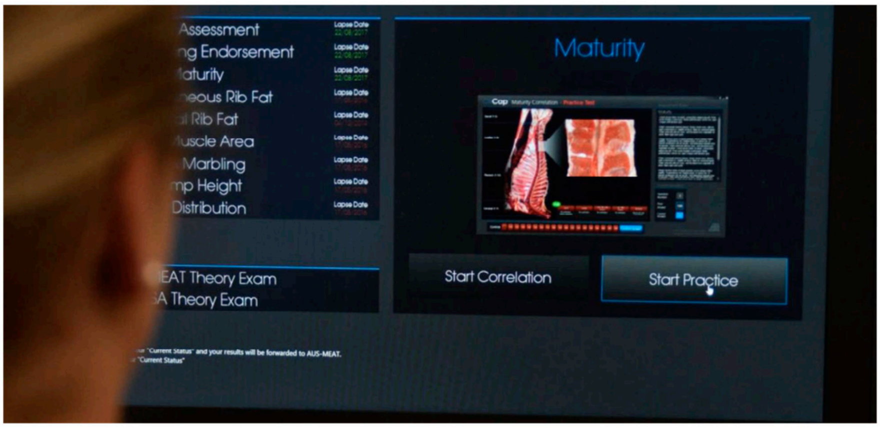

Maturity

a) The variation between the assessment and the standard must not vary on more than 30% of the sample.

b) No assessment may vary by more than two (2) score steps. A score step is defined by the standards.

For example, 14 out of the 20 carcases must be the same as the standard; and the remaining 6 carcases must be within one (1) score step as defined by the standard.

Eye Muscle Area

(a) The variation between the standard and the person being assessed must not be more than ± 4 square centimetres per carcase on more than 10% of the sample.

(b) No assessment may vary by more than ± 8 square centimetres

For example,

on a 10 carcase sample, only one assessment may vary by up to ± 8 square centimetres.

Subcutaneous and Total Rib Fat measurement

(a) The measurement variation between the standard and the person being assessed must not exceed:

1. ± 1mm for all measurements up to and including 5mm;
2. ± 2mm for all measurements over 5mm and up to and including 10mm
3. ± 3mm for all measurements over 10mm

©Copyright, AUS-MEAT Limited. All rights reserved.

OsCap™ correlation requirements

Hump Height & Fat Distribution and Hide Puller Damage

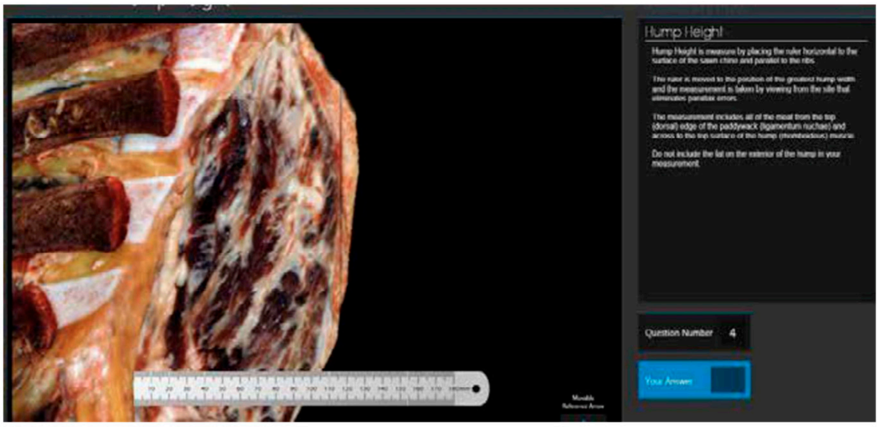

Hump Height

The variation between the assessment and the standard must not vary by more than one (1) score step.

a) A score step is defined as ± 10 millimeters (mm) of the standard.

For example, all carcases used in the sample must be within 10mm of the standard.

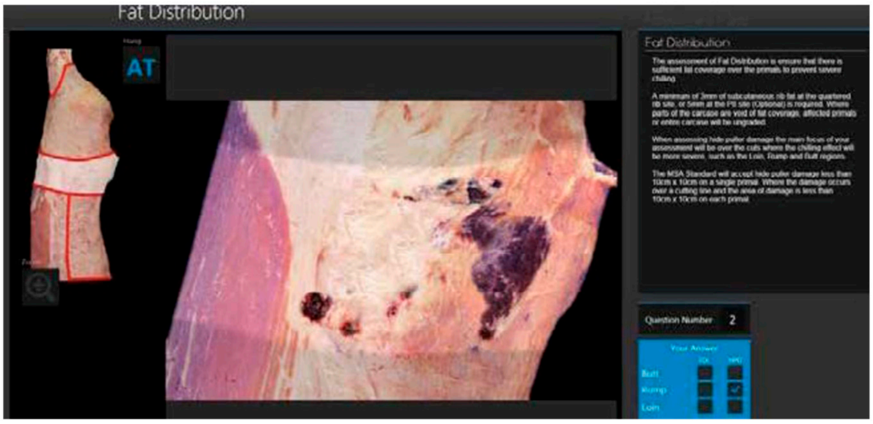

Fat Distribution and Hide puller Damage

There must be no variation between the assessment and the standard.

©Copyright, AUS-MEAT Limited. All rights reserved.

2. **Consumer testing**

Protocols for consumer testing have been previously published (Watson et al., 2008) and are indicated below.

10.1071/EA07176_ AC
©CSIRO 2008
Accessory Publication: Aust. J. Experimental Agric., 2008, 48(11), 1360-1367

Accessory Publication: MSA sensory testing protocols

Detailed work instructions for product collection, sensory sample preparation, cooking and sensory evaluation are presented by Gee *et al.* (2005). These should be adopted as the primary reference for running taste panels. The following summary describes key elements of the protocol for each cooking method to provide an understanding of the process.

Sensory design aspects

Each of the cooking methods described employs common design elements in relation to sensory testing. In MSA experiments prior to 2004 grills were tested utilising a 180 member taste panel with panellists organised in nine sessions of 20 consumers, arranged as three sessions of 20 on three nights of a week. As steaks from a common sample could be cooked on demand this design arrangement worked well and allowed a 5 steak sample to be spread across 5 sessions.

To facilitate automation of operating procedures via software and to apply common sensory design criteria a standard taste panel of 60 consumers testing 36 test samples after 6 first position standard link starter samples was adopted for all cooking methods. The grill design was changed to the 60 consumer format from 2004. The 36 test samples incorporate 6 samples from each of 6 products selected to ensure an eating quality range. In planning any taste panel (pick) the objective is to have minimum eating quality variance between samples within each product and maximum variance between products.

Every consumer is served one sample from each product following the starter link for a total of seven evaluations. While cooking and serving procedures vary to accommodate the particular equipment and characteristics of each method the underlying sensory design is common to all. 10 consumers, treated operationally as five pairs, evaluate each sample tested. The first position link is selected for an expected mid range quality position. Each of the 6 links are served to 5 numerically adjacent pairs. Therefore consumers 1 to 10 eat a common first position product as do 11 to 20 etc.

For the subsequent test samples the 60 consumers are regarded as 5 discreet groups of 12 (six pairs), similar to the original grill protocol of 5 groups of 20. Every sample is tested by 10 consumers (5 pairs). Each pair is allocated from a different subset of 12 (or 20 under the previous grill design) in contrast to the link product. A 6 × 6 Latin

square design of the form below is used to allocate products to each consumer pair with products allocated in the order designated by column.

1	2	3	4	5	6
2	4	1	6	3	5
3	1	5	2	6	4
4	6	2	5	1	3
5	3	6	1	4	2
6	5	4	3	2	1

Therefore, consumer pair one is served a sample of product 1, followed by products 2, 3, 4, 5 and 6 whereas consumer pair two receive products in the order 2, 4, 1, 6, 3, 5 and so on. A new Latin square is commenced for each sub group of 12 consumers. The 5 individual portions (steaks, roast slices, stir-fry strips etc) of each sample are allocated to 5 different order positions as they are dispersed across the 5 subsets of 12 consumers. The net effect is that every sample is tested in 5 of 6 possible different presentational positions by 5 consumer pairs from 5 sub groups.

As there are 6 Latin squares and 6 products, samples from every product occur an equal number of times (6) in each presentational position and before and after each other product. This provides a balance for frequency, order and carryover effects. The 5 pairs who test any one sample are not combined again for any other sample.

Specialist software was developed to control design aspects and collate data from experiments. The software assists in balanced design of product collections and produces unique identification for samples produced. Samples are inventoried together with all available data in a common database. When a taste panel is to be conducted software routines provide for selection of the 36 samples plus links to be tested. The software then allocates each sample according to the design principles

above and produces all associated paper work, plate labels etc. Sensory results are decoded and added to the database by other routines.

Grilling

Sample preparation

The dissected muscle was denuded of all fat and epimysium and a block measuring 75 × 25 × 150 (mm) prepared. If the muscle was large enough to allow multiple locations position within muscle was recorded. Commencing at the anterior, or proximal end of the block five 25mm thick steaks were cut across the grain, using a cutting guide. Each steak was individually wrapped in plastic, placed in a plastic pouch which had been pre-labelled with a unique reference number (EQSRef), a set number used for product storage and other data. Steaks were placed in the pouches in order, thereby retaining a record of their original position in the primal, the pouches vacuum packed and then frozen at the designated days ageing for storage at −18°C.

Picking

MSA software allocated a steak to a pair of consumers and presentational order using the procedures described in sensory design. The software also produced a printed sheet with EQSref numbers for the 10 steaks within each round printed in position for the 7 rounds The frozen sample pouches were opened and the 5 individual frozen steaks from each sample laid out on the pre numbered acetate sheets. When all10 steaks were in position, each sheet and the frozen steaks was placed in another pouch, vacuum packed and stored at −18°C.

Thawing

When required for a taste panel, the 7 frozen round sheets were thawed at 2°–5°C for 24 hours prior to tasting and transported chilled (< 5°C) to the testing site. The bags were opened 1 hour before cooking and the numbered sheet and 10 steaks transferred to a tray for loading onto the griller. Temperature immediately pre-cooking was < 10°C.

Cooking

Steaks were cooked on a Silex clam shell grill unit, set at 220–230°C with the lid set to position #3 or #4 to achieve a 20 to 25 mm gap, the weight set to position 8 and a top plate ratio of 2.75 to ensure even cooking. The griller was switched on 45 minutes prior to cooking and a set of sacrificed starter steaks used to commence the cooking cycle and stabilise temperature recovery. All cooking operations were conducted with reference to a timing schedule to control cooking and serving sequence. Steaks were placed on the Silex in the same order as on the acetate sheet to maintain sample identification. After cooking, steaks were transferred to a cutting board in the same order. Steaks were held for 2 minutes before halving and placing on pre-numbered serving plates. A cross check was conducted by an independent observer confirming the pre printed EQSRef number on the plates matched the round sheet identification. A further check was conducted by confirming a pre-printed label identification on each consumer score sheet against the plate sticker at the point of serving.

3. **Preparation of muscle cuts before and after cooking**

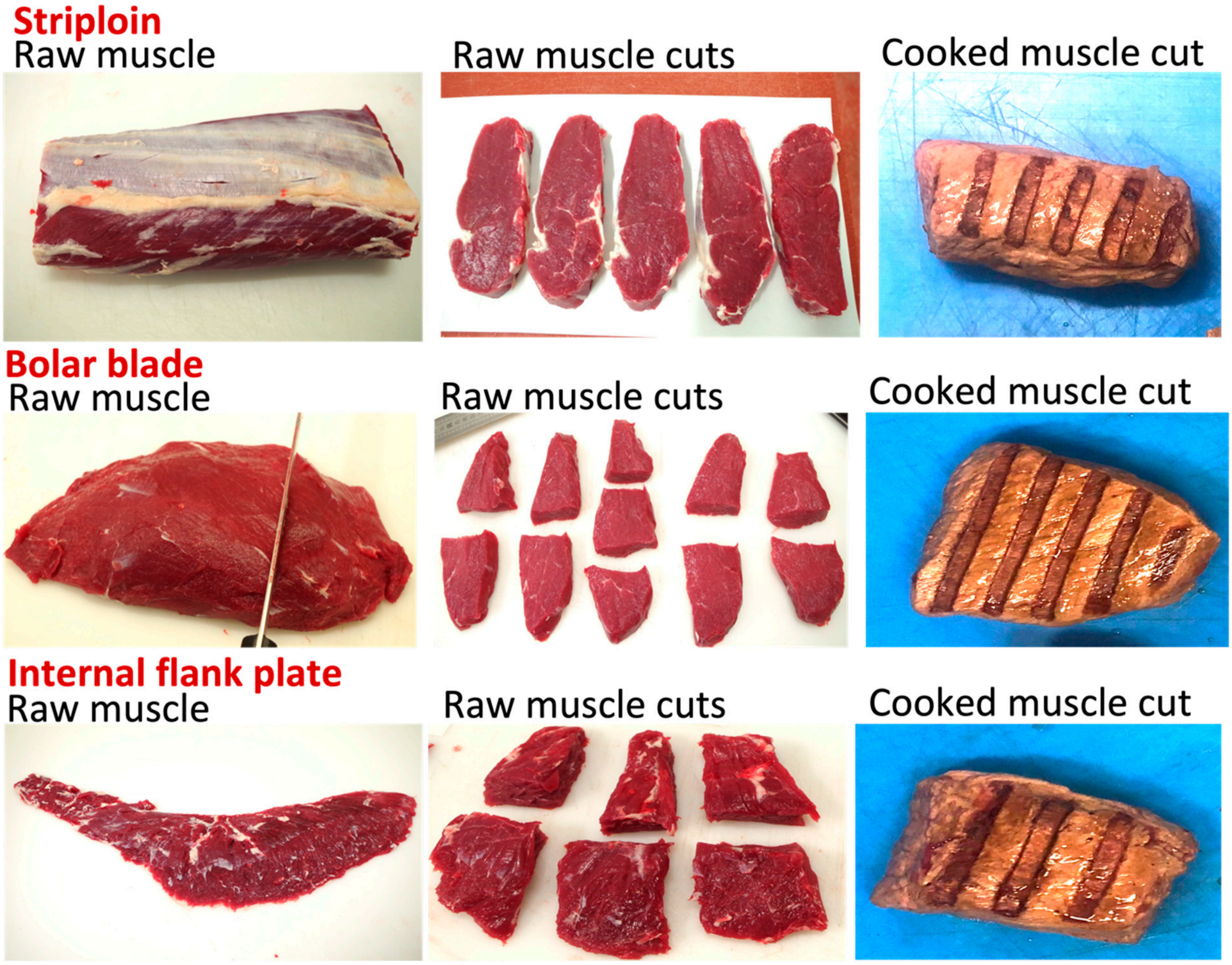

References

AUS-MEAT. (2010). *Australian beef carcase evaluation*. https://www.ausmeat.com.au/WebDocuments/Chiller_Assessment_Language.pdf

Watson, R., Gee, A., Polkinghorne, R.,Porter, M Accessory Publication : MSA sensory testing protocols. *Aust. J. Exp. Agric.* **2008**, *48*, 1360–1367. doi: 10.1071/EA07176_
